# Economic burden of type 2 diabetes in Iran: A cost‐of‐illness study

**DOI:** 10.1002/hsr2.1120

**Published:** 2023-02-20

**Authors:** Habib Jalilian, Somayeh Heydari, Ali Imani, Mozhgan Salimi, Nazanin Mir, Farzad Najafipour

**Affiliations:** ^1^ Department of Health Services Management, School of Health Ahvaz Jundishapur University of Medical Sciences Ahvaz Iran; ^2^ Endocrine Research Center Tabriz University of Medical Sciences Tabriz Iran; ^3^ Health Economics Department, Tabriz Health Service Management Research Center Tabriz University of Medical Sciences Tabriz Iran; ^4^ Health Management and Economics Research Center Iran University of Medical Sciences Tehran Iran

**Keywords:** cost of illness, direct cost, indirect cost, Iran, type 2 diabetes

## Abstract

**Background and Aims:**

Type 2 diabetes mellitus (T2DM) is a prevalent public health problem worldwide, and the economic burden of the disease poses one of the main challenges for health systems in low‐ and middle‐income countries. This study aimed to estimate the economic burden of T2DM in Iran, in 2018.

**Methods:**

This was a cost‐of‐illness study. Three hundred and seventy‐five patients with T2DM who were referred to Imam Reza and Sina's educational and therapeutic centers and Asad Abadi clinic in Tabriz, Iran, in 2018 were included. A researcher‐constructed checklist was used for data collection. Data were analyzed using EXCEL and SPSS software version 22.

**Results:**

Total economic burden of diabetes was estimated at 152,443,862,480.3 (purchasing power parity [PPP], Current International $) (approximately 7.69% of GDP, PPP, Current International $). The mean total direct and indirect costs were 11,278.68 (PPP) (62.35% of mean total cost) and 6808.88 (PPP, Current International $) (37.64% of the total cost), respectively. The mean total direct medical cost and the direct nonmedical cost were 10,819.43 (PPP, Current International $) (59.81% of mean total cost) and 459.24 (PPP, Current International $) (2.53% of mean total cost) per patient, respectively. Besides, the mean direct medical cost was 6.18 times the total per capita expenditure on health, and the total direct medical cost was 8.9% times the total expenditure on health.

**Conclusion:**

Diabetes imposes a substantial economic burden on patients, health systems, and the whole economy. Besides, since the cost of the disease in patients treated with insulin and those with diabetes complications is significantly higher, the reinforcement of self‐care measures and focusing on modifying lifestyle (dietary modification and physical activity) in patients with T2DM can significantly reduce the costs of the disease.

## 1 INTRODUCTION

Diabetes is the fourth most prevalent noncommunicable disease that kills 1.6 million people each year across the world and is one of the most important public health challenges to both developing and developed countries.^1^ In 2021, approximately 537 million adults (20–79 years) were living with diabetes. The total number of people living with diabetes is predicted to soar to 643 million by 2030 and 783 million by 2045. Three in four adults with diabetes were living in low‐ and middle‐income countries.^2^ WHO projects that the number of people with diabetes reaches 552 million by 2030, and diabetes will be the seventh leading cause of death in 2030.^3^, ^4^ There are more than 32 million patients with diabetes in the Middle East (ME); this figure is expected to double by 2040.^5^ According to the latest International Diabetes Federation (IDF) (January 16, 2023), type 2 diabetes mellitus (T2DM) is the most common type of diabetes, making up around 90% of all diabetes.^6^ In 2021, the global prevalence of T2DM in adults was 536.6 million people (10.5%), and there would be 783.2 million people (12.2%) living with diabetes worldwide by 2045.^7^

Diabetes imposes a substantial economic burden on countries, health systems, people with diabetes, and their families. According to the IDF report in 2021, diabetes caused at least USD 966 billion in health expenditure (9% of total spending on adults),^2^ and the economic burden as a percentage of GDP was more considerable in middle‐income countries than in high‐income countries.^8^

Iran, as one of the countries of the Middle East and North Africa (MENA) region, has been affected by the diabetes epidemic.^9^, ^10^ In 2020, the prevalence of diabetes and the total cases of diabetes in adults were estimated to be 9.4% and 5,387,200, respectively.^11^ In Iran, in 2018, approximately 5.3 million people were diagnosed with diabetes.^12^ It is projected that 9.2 million people in Iran will have Diabetes by 2030.^13^ This continuous and notable rise in the prevalence of diabetes shows the burden of disease in Iran, particularly when considering the effect of diabetes‐related complications.^13^

The global prevalence of diabetes and the economic and social burden of diabetes is expected to rise due to changing demographics and lifestyle, especially in middle‐ and low‐income countries.^14^ Diabetes and its multidimensional implications include large financial resources for patients and national economies owing to the direct and associated costs of treatment and indirect costs related to loss of work and wages.^15^ In 2017, global healthcare expenditure for the management of diabetes and its complications among people with diabetes 18–99 years old was estimated at USD 850 billion, and this figure is predicted to increase by 7% to USD 958 billion in 2045.^16^ In Iran, in 2021, diabetes‐related health expenditure per person, USD was estimated at 1354.8 USD. The figure is expected to rise to 1663.5 USD by 2030 and 1839.2 USD per person by 2045.^17^

Given the increasing prevalence of T2DM in Iran and the effects of type 2 diabetes costs on patients, third‐party payer organizations, and the healthcare system, it is crucial for health policymakers to know how much costs are imposed on the health system because of diabetes. Knowing the economic burden of diabetes can help policymakers make better decisions in prioritizing cost‐effective measures. Thus, the principal objective of this study was to estimate the economic burden of type 2 diabetes in Iran, in 2018.

## 2 METHODS

### 2.1 Study design

This prevalence‐based cost of illness study was conducted from the societal perspective using bottom‐up approach costing.

### 2.2 Study setting and population

The statistical population included all patients with type 2 diabetes in East Azerbaijan province referring to Imam Reza and Sina's educational and therapeutic centers and Asad Abadi clinic in Tabriz, Iran, in 2018. The population of East Azerbaijan province is estimated at 4 million, and the rate of diabetes incidence is 10%. So, the population of patients with diabetes is estimated at 40,000, and based on the Morgan table, 375 patients with T2DM were selected. Hence, based on the calculated population size and incidence rate, the estimated population is estimated at 40,000. Due to the inaccessibility to all of the statistical population in our study, the convenience sampling method was used consecutively.

A researcher‐made checklist was used for data collection. The checklist design process was as follows: interview with endocrinologists (*n* = 7), interview with researchers who had conducted at least one cost of illness study (*n* = 3), interview with professors in Health Economics (*n* = 2), interview with T2DM patients (*n* = 15), and the review of records of inpatients and outpatients of T2DM patients. The checklist consists of demographic variables (age, gender, supplementary insurance status, and the type of basic insurance), duration of the disease, treatment type, and questions about diabetes‐related costs. The questionnaires were completed during a 20‐min face‐to‐face interview by one of the research team.

### 2.3 Cost classification and definition

In this study, the economic burden of diabetes was estimated by calculating direct medical costs, direct nonmedical costs, and indirect costs for a year. Direct medical costs were expenditures related to medical services and drugs associated with diagnosis and treatment performed in hospitals and clinics. Direct nonmedical costs included commuting costs and costs associated with accommodations and extra nutrition for patients with diabetes during the period of treatment. Indirect costs were defined as days of lost productivity for both patients and their family members caused by outpatient visits and hospitalization due to diabetes. Moreover, data related to outpatient costs, direct nonmedical costs, and missed workdays were collected once every 2 months for a year using the checklist.

### 2.4 Cost estimation

In the present study, data related to the hospitalization part of direct medical costs were extracted from patients’ records, and outpatient parts of direct medical costs and direct nonmedical costs were obtained via an interview with patients and their families, respectively.

Indirect cost was estimated based on the Human Capital Approach via GDP per capita (2018), lost productivity due to missed workdays, and premature death due to diabetes. First, to estimate the cost of missed workdays per patient, we calculated the annual average number of missed workdays of the patients and their families due to diabetes and then multiplied it by the GDP per capita; in this way, we estimated the cost of missed workdays per patient. To calculate the cost of premature death due to disease, the average duration of premature death was multiplied by GDP per capita (Formulas (1) and (2)):

*Formula (1): Lost productivity due to missed workdays of patients and their families* = *(the number of missed workdays of patients and their families/the number of workdays of the year)* × *GDP per capita (PPP, Current International $)*.

*Formula (2): (retirement age in Iran − the median age of death)* × *GDP per capita* (PPP, Current International $).

All costs in this study were adjusted to current international dollars based on the World Bank's PPP conversion factors (https://data.worldbank.org/indicator/NY.GDP.PCAP.PP.CD). A detailed description of unit costs is presented in Table 1.

**Table 1:** Cost categories and sources of applied unit costs.

Sector	Service/goods	Data source	Units	Monetary values (unit costs)
Surgery costs	Operating room consumables and equipment, operating room medication, physician (surgeon) work, anesthetic	Medical records	Relative value unit/current procedural terminology	Medical tariffs
Hoteling costs	Cost of nonphysician human resources, depreciation, repairs and maintenance, food, energy, other goods and services not included in the billing separately	Medical records	Days of hospital stay	Reimbursement schedule
Visit costs	Visit in hospital OR physician's office	Medical records/questionnaire	Quantity	Reimbursement schedule/Medical tariffs
Medication costs	Medications that are recorded with a separate title in hospital billing codes/medicines that the patient purchase outside the hospital	Medical records/questionnaire	Quantity	Reimbursement schedule
Nursing services cost	The cost of nursing care, and the cost of all the care for hospitalized patients	Medical records	Quantity	Medical tariffs
Laboratory cost	Fasting blood sugar test, glucose tolerance test, random blood sugar test, glucose screening test, and glucose tolerance test	Medical records/questionnaire	Quantity	Reimbursement schedule
Insulin costs	Insulin, insulin syringes, insulin pens, insulin pump, injections, and drug cost	Questionnaire	Quantity	Reimbursement schedule
Medical equipment's cost	blood sugar meters, blood lancets, diabetic test strips, ketone test strips, glucose tablets and glucagon, diabetes medical alert bracelet	Questionnaire	Quantity	Reimbursement schedule
Physiotherapy cost	Costs that patients pay for receiving physiotherapy services	Medical records/questionnaire	Quantity	Reimbursement schedule
Dietetics cost	Costs that patients pay for adhering to a specific diet	Questionnaire	Quantity	Consumer price
Traditional medicine cost	Complementary treatments of traditional medicine from informal providers	Questionnaire	Quantity	Consumer price
Nutritionist consultation cost	Dietitian's consultation cost	Medical records/questionnaire	Quantity	Reimbursement schedule/medical tariffs
Commuting and food costs	Costs that patients and caregivers incurred due to commuting to treatment centers	Questionnaire	Quantity	Consumer price
Accommodation costs	Costs that patients and caregivers incurred due to residing in a hotel or hostel for receiving services in other cities	Questionnaire	Quantity	Consumer price
Morbidity costs	Cost of lost productivity due to the missed workdays	Questionnaire	Days	The current average wage in the country
Mortality cost	Productivity loss due to premature death	Questionnaire	Years	The current average wage in the country

### 2.5 Data analysis

Data analysis was performed using EXCEL and SPSS version 22. Descriptive statistics (mean and SD) were used for cost estimates. Spearman correlation coefficient (CC) was used to examine the association between cost items and age, duration of the disease, the number of hospitalization, and Mann–Whitney for the association between mean costs and gender, informal payment, basic insurance coverage, supplemental insurance coverage, and habitation status. Kruskal–Wallis for the association between mean costs and age, Insurance type, treatment type, and cause of hospitalization. The tests were carried out at a 5% significance level, and a *p‐value* ≤ 0.05 was considered as significant.

### 2.6 Ethics approval and consent to participate

This study was approved by the Ethics Committee of Tabriz University of Medical Sciences (Reference No: IR.TBZMED.REC.1395.672). All participants were informed about the purpose of the study and assured of confidentiality and anonymity. Moreover, written informed consent was taken from each of them before the total procedure. All methods were carried out in accordance with the relevant guidelines and regulations.

## 3 RESULTS

A total of 375 patients with T2DM participated in this study. Most of the respondents (50.2%) were male. The mean (SD) age of patients, disease duration, and the number of hospitalization were 60.73 ± 14.15, 11.32 ± 8/03 year, and 1.75 ± 0.87, respectively. 98.1% of patients were covered by basic insurance, and only 13.1% of them were covered by supplemental insurance (Table 2). The results of Mann–Whitney and Kruskal–Wallis tests showed total medical direct costs were significantly associated with informal payments status, basic insurance coverage status, type of health insurance, habitation status, the cause of hospitalization, type of treatment, and direct medical costs (Table 1). Also, there was a significant correlation between age (*p* = 0.01, CC = 0.13), disease duration (*p* = 0.02, CC = 0.18), hospitalization number (*p* = 0.02, CC* = 0.14), and total medical direct costs. With an increase in age, disease duration, and hospitalization number, the total medical direct costs would increase (Table 2).

**Table 2:** Frequency (%) of demographics and clinical variable and median (interquartile range) of total medical direct costs.

Variables	Modes	Frequency (%)	Median (interquartile range) (PPP, Current International $)	*p* value
Gender	Male	180 (50.2)	7921.82 (14,626.19)	0.19
	Female	177 (49.8)	7498.27 (7233.76)	
Informal payment	Yes	45 (18.1)	12,715.86 (17,917.92)	0.02*
	No	204 (81.9)	8707.49 (11,418.16)	
Age groups	<40	33 (10.3)	5654.87 (5246.50)	0.10
	40–60	124 (38.8)	8367.75 (13,772.32)	
	>60	163 (50.9)	7712.70 (9768.58)	
Basic insurance coverage	Yes	350 (98.1)	7181.93 (9267.11)	0.01*
	No	7 (1.9)	960.32 (12,357.26)	
Insurance type	Iranian health insurance	159 (50.5)	9429.29 (13,399.67)	0.03*
	Social security	135 (42.9)	7120.54 (8816.43)	
	Others	21 (6.7)	7536.29 (7918.72)	
Supplemental insurance coverage	Yes	47 (13.1)	7754.78 (16,176.32)	0.65
	No	310 (86.9)	7712.70 (9774.43)	
Habitation status	Patients living in urban areas	234 (65.6)	7159.48 (9574.03)	<0.001**
	Patients living in rural areas	123 (34.4)	8846.23 (15,092.24)	
Treatment type	Insulin	244 (68.4)	8852.47 (11,099.68)	<0.001**
	Oral medication	102 (28.7)	6923.51 (8600.77)	
	No medication	11 (2.9)	2427.18 (2822.58)	
Cause of hospitalization	Foot ulcer	79 (22)	14,680.66 (21,345.02)	0.01*
	Nephropathy	60 (16.7)	7317.38 (8719.51)	
	Neuropathy	35 (9.8)	8647.82 (12,208.88)	
	Retinopathy	29 (8.2)	7210.54 (7045.18)	
	Other reasons	154 (43.3)	6882.39 (7528.60)	

Abbreviation: PPP, purchasing power parity.

*p < 0.05; **p < 0.001.

According to the results of Table 3, the mean direct medical costs of patients with no history of hospitalization were 9757.01 (PPP, Current International $) (approximately 813.08 [PPP, Current International $] per month), and those with a history of hospitalization incurred an additional cost of 3517.97 (PPP, Current International $) in addition to this costs. The highest amount of outpatient costs were related to insulin and wound healing costs at 3087.35 (PPP, Current International $) and 1794.46 (PPP, Current International $) per patient, respectively. Hoteling and medicine costs accounted for the highest amount of hospitalization costs at 1160.37 (PPP, Current International $), and 641.90 (PPP, Current International $) per patient, respectively (Table 2). The contribution of basic insurance, patient, subsidy, supplementary insurance, and hospital discount in the reimbursement of hospitalization costs was 82.54%, 7.25%, 6.95%, 2%, and 1.22%, respectively.

**Table 3:** Outpatient and hospitalization costs of type 2 diabetes.

Cost items	Mean (PPP, Current International $)	*SD*
Outpatient costs		
Insulin cost	3087.35	7732.58
Wound healing cost	1794.46	5503.52
Medicine cost	1721.00	3008.61
Specialist physician visit cost	552.65	959.46
Physiotherapy cost	520.00	1734.45
Diagnostic cost	428.93	1139.66
Medical equipment's cost	417.08	727.74
Paraclinical cost	382.42	638.31
Dietetics cost	314.65	677.53
Ancillary equipment cost	209.48	469.35
General physician visit cost	153.92	341.17
Traditional medicine cost	107.81	487.13
Nutritionist Consultation cost	66.97	217.04
Total outpatient cost	9757.01	23636.83
Hospitalization costs		
Hoteling cost	1160.37	1646.82
Medicine cost	641.90	1140.88
Visit cost	600.35	964.24
Surgery cost	271.60	910.37
Laboratory cost	181.64	181.57
Diagnostic cost	169.77	202.99
Supplementary cost	160.41	374.77
Patient relatives cost	76.60	131.50
Nursing services cost	67.21	93.30
Injection cost	66.34	182.73
Dialysis cost	40.64	371.93
MRI and CT scan cost	31.09	80.11
Hospital services cost	30.93	138.09
Sanitary bags cost	8.303	11.44
Ambulance cost	7.80	21.68
Rehabilitation cost	2.65	15.06
Total hospitalization cost	3517.97	4693.05

Abbreviation: PPP, purchasing power parity.

Table 4 reports the proportion of cost components from the total cost of type 2 diabetes. Total direct medical costs to the T2DM patients (all inpatients and outpatients) in the study were 10,819.43 (PPP, Current International $) per patient, making up 59.99% of the mean total costs. For outpatients, the mean total direct medical costs, the mean total nonmedical direct costs, and the mean total indirect costs accounted for 58.19%, 2.23%, and 39.57% of mean total costs, respectively. For inpatients, the mean total direct medical costs, the mean total nonmedical direct costs, and the mean total indirect costs accounted for 63.27%, 3.17%, and 33.54% of total mean costs.

**Table 4:** Proportion of cost components from the total cost of type 2 diabetes.

Cost items	Inpatients		Outpatients	
	Mean cost (PPP, Current International $)	% of the total cost	Mean cost (PPP, Current International $)	% of the total cost
Medical direct cost				
Mean outpatient cost	9757.01	46.5	9757.01	58.19
Mean hospitalization cost	3517.97	16.76	0	0
Total mean medical direct cost	13,274.98	63.27	9757.01	58.19
Nonmedical direct cost				
Mean commuting cost	471.43	2.24	374.64	2.23
Mean accommodation and food cost	194.49	0.92	0	0
Total mean Nonmedical direct cost	665.95	3.17	374.64	2.23
Total mean direct cost	13,940.97	66.45	10,131.65	60.42
Indirect cost				
Mean cost of lost productivity due to the missed workdays	6955.56	33.15	6552.32	39.07
Mean mortality cost (for all inpatients and outpatients)	134.78	0.39	134.78	0.49
Total mean indirect cost	7090.34	33.54	6687.1	39.57
Total cost	20,978.92	100	16,766.46	100

Abbreviation: PPP, purchasing power parity.

The lost productivity due to missed workdays of patients and their families was estimated via formula (1):

*Formula (1): Lost productivity due to missed workdays of patients and their families* = *(the number of missed workdays of patients and their families/the number of workdays of the year)* × *GDP per capita*.

*Lost productivity due to missed workdays of patients and their families* = *0.3711* + *0.1341* = *0.5052* × *14,535.9* = *7343.53 PPP, Current International $*.

*Lost productivity due to missed workdays of patients and their families (patients residing in urban areas) = 0.3292* + *0.1327* = *0.4619* × *14,535.9* = *6714.13 PPP, Current International $*.

*Lost productivity due to missed workdays of patients and their families (patients residing in rural areas) = 0.4311* + *0.1376* = *0.5687* × *14,535.9* = *8266.56 PPP, Current International $*.

- *Estimating total costs*

Total patients with T2DM in Iran = 8,428,105

Hospitalization rate = 30.2%

Outpatient rate = 0.698

1. *Total medical direct costs of diabetes in Iran = (the number of patients with T2DM in Iran* × *mean total outpatient costs)* + *((inpatient rate)* × *the number of patients with T2DM in Iran* × *mean total inpatient costs) = (8,428,105* × *9757.01)* + *(0.302)* × *8,428,105* × *3517.97)* = *82,233,104,766.05* + *8,954,245,805.414* = *91,187,350,571.19 (PPP, Current International $)*.
2. *Total nonmedical direct costs of diabetes in Iran* = *([outpatient rate] the number of diabetic* × *mean nonmedical direct costs of outpatient diabetic)* + *([inpatient rate] the number of diabetic* × *mean nonmedical direct costs of inpatient diabetic)* = *([0.698] 8,428,105* × *374.64)* + *[(0.302) 8,428,105* × *665.95]* = *2,175,521,122.21 + 1,695,034,350.47* = *3,870,555,472.68 (PPP, Current International $) 95,057,906,043.87*.
3. *Total indirect costs of diabetes in Iran* = *total mortality costs* + *total morbidity costs* = *1,135,953,666.66* + *56,250,002,769.77* = *57,385,956,436.43 (PPP, Current International $)*.

3.1 *Total mortality costs* = *total number of death* × *mean lost years of life (premature years)* × *GDP per capita PPP, Current International $* = *12,660* × *10* × *14,535.9* = *1,840,244,940*

PV = FV/(1 + *i*) ^*N*^

where *i* = 0.05 *and N* = 10.

*Discounted total mortality costs* = *Future value/discount factors* = *1,840,244,940/1.62* = *1,135,953,666.66*.

3.2 *Total morbidity costs = (the number of patients with T2DM in Iran* × *(0.302)* × *mean lost productivity costs of inpatients)* + *(the number of patients with T2DM in Iran* × *(0.698)* × *mean lost productivity costs of outpatients)* = *(8,428,105* × *0.302* × *6955.56)* + *(8,428,105* × *0.698* × *6552.32)* = *17,703,901,384.16* + *38,546,101,385.61 = 56,250,002,769.77*.

4. *Total economic burden of diabetes in Iran* = *total medical direct costs* + *total nonmedical direct costs* + *total indirect costs* = *91,187,350,571.19* + *3,870,555,472.68* + *57,385,956,436.43* = *152,443,862,480.3 (PPP, Current International $)*.

- *Cost components as a percentage of total costs*
*Total medical direct costs as percentage of total costs* = *total medical direct costs/total costs* = *91,187,350,571.19/152,443,862,480.3* = *0.59*.
*Total nonmedical direct costs as percentage of total costs* = *Total nonmedical direct costs/total costs* = *3,870,555,472.68/152,443,862,480.3* = *0.02*.
*Total indirect costs as percentage of total costs* = *total indirect costs/total costs* = *57,385,956,436.43/152,443,862,480.3* = *0.37*.
- *Medical direct costs as a percentage of health expenditure*

Total per capita expenditure on health in Iran (2017) = 1748.40 (*PPP, Current International $)* (https://data.worldbank.org/indicator/SH.XPD.CHEX.PP.CD).

Total Health Expenditure in Iran (2017) = 1,015,528,323,000 (*PPP, Current International $)*.

Total GDP (PPP, Current International $) of Iran (2017) = 1,172,665,500,000.

https://data.worldbank.org/indicator/NY.GDP.MKTP.PP.CD

GDP per capita, PPP (current international $) (World Bank, 2017) = 14,535.9 (PPP, Current International $).

https://data.worldbank.org/indicator/NY.GDP.PCAP.PP.CD?end=2018&locations=MW.&start=1960

*Mean direct Medical costs of inpatients/Total per capita expenditure on health* = *13,274.98/1748.40* = *7.59*.

*Mean direct Medical costs of outpatients/Total per capita expenditure on health* = *9757.01/1748.40* = *5.58*.

*Mean medical direct costs (for all inpatients and outpatients)* = *outpatient costs* + *((inpatient rate)* × *inpatient costs* = *9757.01* + *((0.302)* × *3517.97* = *10,819.43*.

*Mean direct medical costs (for all inpatients and outpatients)/Total per capita expenditure on health* = *10,819.43/1748.40* = *6.18*.

*Total medical direct costs/total health expenditure* = *91,187,350,571.19/1,015,528,323,000* = *0.089* = *8.9%*.

*Total costs (PPP, Current International $)/total GDP (PPP, Current International $)* =  *152,443,862,480.3/1,172,665,500,000* = *7.69%*.

## 4 DISCUSSION

In this study, a total of 375 patients with type 2 diabetes were studied. The total economic burden was 18,087.56 PPP, Current International $ ($US 6877.39) per patient, representing 1.24 times GDP per capita (GDP per capita, PPP, Current International $) (2017) = 14,535.9 (PPP, Current International $). Our study showed that direct medical costs were the major component of the total economic burden of patients with T2DM. The mean total direct medical cost was estimated at 10,819.43 (PPP, Current International $) per patient, representing 74.43% of GDP per capita. Diabetes is a chronic disease that requires consistent healthcare, and people with T2DM may spend years with the disease and its complications. Therefore, in contrast to acute and fatal diseases, most of the economic burden of this disease is related to direct medical costs.

Although the results of some previous studies are almost similar to the results of the present study in some cases, the estimated economic burden of the disease differs according to the design and methods of the studies. The reason for these differences can be attributed to methodological differences and a difference in the quantity and quality of services provided to patients with T2DM in some areas, differences in currencies, and the timing of studies. So, it is difficult to compare results across studies because they were assessed with different currencies.

Nguyen Tu et al., in Vietnam, conducted a cross‐sectional study and analyzed 392 patients with T2DM. The authors showed that the annual cost was $24,610 per patient, equivalent to 12% of GDP per capita. The highest amount of cost was direct medical cost at $12,730, and pharmaceuticals constituted 27.5% and 53.2% of the total cost and total medical direct cost, respectively.^13^ Seuring et al. conducted a systematic review of the global evidence on the costs of type 2 diabetes from 2001 to 2014. Their review demonstrated that diabetes imposed a significant burden on countries and accounted for a significant proportion of GDP. They reported direct costs were higher than indirect costs. Their regression analysis showed that direct diabetes costs were closely and positively associated with the GDP per capita of countries. The United States stood out as having particularly high costs, even after controlling for GDP per capita.^15^ In a study by Migdalis et al., in Greece, the total annual cost of diabetes was €7111 per patient.^18^ In the study of Shuyu et al. in Singapore, the mean annual medical direct cost was $2034, and inpatient services constituted the highest percentage of total medical direct cost at 61%, outpatient services and accident and emergency services were next at 35%, and 4% of total medical direct cost, respectively.^19^ Their results are almost similar to the present study. In 2014, in Nigeria, the direct cost of type 2 diabetes was $28,457 per patient,^20^ which is higher than our study. In 2015, on average, the annual cost of treating one case of diabetes mellitus (DM) in Latin America and the Caribbean was estimated between US $1088 and US $1818. Per capita, National Health Expenditures were estimated at US $1061.^21^

Of the direct medical costs, the proportion of outpatient costs to the total economic burden of diabetes was 2.77 times higher than inpatient costs (9757.01 vs. 3517.97). Outpatient costs occur most frequently in any of the patients with T2DM, while hospitalization costs occur only in some patients with T2DM. Hence, much of the direct medical costs were due to the costs of outpatient.

In this study, the mean outpatient cost was estimated at 9757.01 (PPP, Current International $), accounting for 53.94% of the mean total cost. Insulin and wound healing costs were the most costly contributor to outpatient costs, with 3087.35 (PPP, Current International $) (31.64% of the outpatient cost and 17.06% of mean total cost) and 1794.46 (PPP, Current International $) (18.39% of the outpatient cost and 9.92% of mean total cost), respectively. But, in a study by Barcelo et al., in Latin America and the Caribbean, insulin was the second‐highest estimated cost after treatment of complications (between $6 and $11 billion).^21^ In our study, hospitalization costs were estimated at 3517.97, making up 32.51% of direct medical costs and 19.44% of the total cost. In a study by Henriques et al., in Brazil (2012–2014), the median hospitalization cost was $3656 per patient, equivalent to 53.1% of total costs.^22^ An Italian study assessed the management cost of patients with T2DM and demonstrated the highest amount of costs of T2DM were attributed to hospitalization costs, both insulin‐dependent, and insulin‐independent, representing 51% (€1079.20) and 57% (€335.57) of the total costs, respectively.^23^ In a study by Farshchi et al., in Iran, 1000 patients with type 2 diabetes were analyzed for 10 years. The average para clinic costs and inpatient costs were 3936 ± 47.8 and 15,207 ± 1045 per patient, respectively.^24^

In this study, different variables such as the history of informal payment, the incidence of complications, insurance coverage, type of insurance, type of treatment received, and region of residence affected the direct costs of diabetes. The direct medical costs among those undergoing insulin treatment and oral medications were, respectively, 3.64 and 2.85 times higher than those who did not take any medicine and just relied on modifying their lifestyle (diet and physical activity) to control their disease. Hence, it appears that adequate focus on self‐care measures and modifying lifestyle in T2DM patients can lead to a significant decrease in disease costs, in addition to improving clinical outcomes.

Direct medical costs in patients with a foot ulcer, neuropathy, nephropathy, and retinopathy were 2.13, 1.25, 1.06, and 1.04 times higher, respectively than in other patients. Patients with foot ulcers and neuropathy (neurological complications) incurred the highest costs. It seems that the incidence of diabetes complications increases the disease costs by increasing the need for more complicated treatment care and admission to the hospital, in addition to a reduction of quality of life and increased mortality and disability in T2DM patients. So, to manage the costs of patients with type 2 diabetes, health policymakers should focus more on preventive and self‐care measures and improvement of service quality in the first and second levels of the referral system. Also, integrated personalized diabetes management can be a solution to reduce complications and improve treatment outcomes.^25^ It has been shown that the cost of complications is the primary driver of healthcare expenditure for type 2 diabetes.^26^, ^27^ A study in Latin America and the Caribbean demonstrated that treatment of complications made up the highest amount of direct medical cost.^21^ Migdalis et al. in Greece showed management of co‐morbidities, pharmaceutical treatment, and diabetes treatment per patient accounted for 48%, 35.9%, and 14.95% of the costs of diabetes, respectively. They also showed that co‐morbidities constituted the highest percentage of diabetes costs at 52.6%, and diabetes medication cost was next with 14.3% for controlled patients.^18^

According to the results, 18.1% of patients had informal payment, and direct medical costs among patients who incurred informal payment were 1.46 times higher than those without informal payment. These payments are made by patients to better access healthcare services. Informal payments are most of the time regarded as a sign, indicating a wide range of underlying problems in the healthcare system. It is representing of inefficiencies of the health system in providing equitable access to health services and imposes an additional economic burden on patients. Hence, appropriate control measures should be taken to prevent receiving informal payments by physicians.

Direct medical costs among patients under health insurance coverage were 7.47 times as much as those who were not under the coverage of health insurance. It seems that those who were not under the coverage of health insurance had poor financial access to healthcare services and were more likely to be deprived of receiving health services. Although equitable access to health services is considered a fundamental human right regardless of ability to pay in Iran, 1.9% of patients with T2DM are not covered by health insurance, and these patients are deprived of receiving essential services due to their inability to pay for medical costs. Therefore, it is necessary to identify these patients and take the necessary supportive measures to improve their access to essential healthcare services and financial protection of these patients against the catastrophic costs incurred by the disease by the government, health insurance organizations, charities, and other related institutions.

The cost of the disease was more likely to be higher among those under Iranian health insurance coverage than those under social security insurance. This difference may be due to two reasons: first, the Social Security Administration may have been more successful in managing the costs of diabetes, and second, Iranian health insurance may have provided more services to these patients. However, without sufficient information about the quantity and quality of services provided to patients covered by these two insurance organizations, as well as information about the quality of life‐related to healthcare and clinical outcomes of these two groups, a definite judgment is not possible, and needs further investigation.

Direct medical costs among patients residing in rural areas were 23% higher when compared with those residing in urban areas. Most patients living in rural areas who were referred to urban healthcare centers had a more complicated situation, and they need more advanced and expensive treatment care, which may imply that patients residing in rural areas were more likely to delay or not receive needed medical care due to the long distance from medical centers and limited access to the tertiary level of health services and have more complex and expensive services need.

The mean direct nonmedical cost in our study was 459.24 (PPP, Current International $) per patient (2.54% of the mean total cost). Commuting costs accounted for the highest direct nonmedical costs. In 2016, in Vietnam, the estimated nonmedical direct cost was $34.40.^13^

The mean indirect cost in this study was 6808.88 (PPP, Current International $) per patient, accounting for 37.64% of the mean total cost (morbidity costs, 36.89%; mortality costs, 1.20%). In Vietnam, in 2016, the indirect cost of T2DM accounted for 34.3% of the total cost.^13^ Bommer et al., in 2015 conducted a study to estimate diabetes‐related costs in adults aged 20–79 years in 184 countries and reported that indirect costs accounted for 34.7% of the total burden.^8^ Marcellusi et al., in 2014, studied 2.5 million patients with DM in Italy and reported 54% of diabetes‐related costs were related to indirect costs (absenteeism and early retirement), and 46% were related to direct costs (drugs, hospitalization, monitoring, and adverse events).^28^ Javanbakht et al., in 2009, conducted a prevalence‐based cost‐of‐illness (COI) to assess the economic burden of T2DM in Iran. In their study the total national cost of T2DM was 3.78 billion USD in Iran, of which 2.04 billion USD was for direct medical costs and direct nonmedical costs and 1.73 billion USD was for the indirect cost (temporary disability 19.3%, permanent disability 26%, premature mortality 54.7%).^9^ Ulrich et al. showed that individuals with type 2 diabetes had 1.81 times higher direct and 2.07 times higher indirect annual costs than those without diabetes.^29^ In a study by Bayati et al., in 2008, the highest percentage of indirect cost was attributed to premature mortality at 58.5%, disability cost, and missed workdays were next at 30.3% and 11.2%, respectively.^25^ In 2015 over 41 million adults aged 20 years and over were estimated to have T2DM in Latin America and the Caribbean. The total indirect cost of diabetes was US $57.1 billion, of which US $27.5 billion was due to premature mortality, US $16.2 billion to permanent disability, and US $13.3 billion due to temporary disability. The total direct cost ranged from US $45 to US $66 billion, of which the highest cost was due to the treatment of complications (US $1616 to US $26 billion).^21^

Direct nonmedical costs especially, commuting costs, were higher for those living in rural areas. Also, the mean cost of lost productivity was higher for patients living in rural areas (8266.56 [PPP, Current International $]) than those living in urban areas (6714.13 [PPP, Current International $]). It has meant that they spent more time and had a higher number of missed workdays. It seems that despite all the efforts made by the Ministry of Health of Iran to achieve equitable access to health services, financial, physical, and temporal access to health services is still largely unfair. This disparity is remarkable; hence, policymakers should adopt supportive measures for those residing in rural areas or away from advanced treatment centers.

In our study, mean direct medical costs (for all inpatients and outpatients) were 6.18 times the total per capita expenditure on health. This suggests that the health expenditure of each patient with T2DM is more than six times as high as the average health expenditure of other people. The total medical direct costs of diabetes were equivalent to 8.9% of total health expenditure. This has meant that diabetes requires prevention measures and some interventions should be taken into consideration in the health system program.

The contribution of basic insurance, patient, subsidy, supplementary insurance, and hospital discount in the reimbursement of hospitalization costs was 82.54%, 7.25%, 6.95%, 2%, and 1.22%, respectively. Moreover, the patient contribution to the total outpatient cost was estimated at 44.44%. The patient contribution of total medical direct costs was 0.36. Except for hospitalization costs, health insurance does not provide adequate support for people with T2DM, and these patients incurred a significant share of outpatient costs. Although these people pay these costs gradually over time, the share of people with T2DM in reimbursing outpatient costs puts significant economic pressure on them, especially in low‐income and vulnerable groups. Therefore, it is necessary for the government to revise the services coverage and financial coverage for these patients and provide more support for them. Besides, strengthening the role of supplementary insurance can also be considered as an adjuvant solution. Currently, private insurance in Iran plays a minor role in reimbursing costs. A systematic review showed that health insurance coverage could have protective effects against out‐of‐pocket expenditures, but mainly for those with higher incomes, while the poor often lacked coverage.^30^, ^31^, ^32^ Another systematic review demonstrated that, once people were covered by health insurance, their risk of incurring catastrophic expenditures decreased significantly.^33^

## 5 LIMITATIONS

This study has some limitations. The cost of decreased productivity at work due to disability and its complications and loss of income due to job changes caused by diabetes and its complications have not been estimated.

### 5.1 Policy implications

Diabetes imposes a substantial economic burden on patients, health systems, and the whole economy. Besides, since the cost of the disease in patients treated with insulin and those with T2DM complications is significantly higher, the reinforcement of self‐care measures and focusing on modifying lifestyle (dietary modification and physical activity) in patients with T2DM can significantly reduce the costs of the disease. Since the cost of the disease for those living in rural areas and those who were forced to refer to the tertiary level treatment centers in large cities was higher than for those living in urban areas, more financial and practical support should be provided for these people by the government. Also, due to the high rate of patients’ copayment, especially in the reimbursement of outpatient costs, it is necessary to strengthen the role of health insurance in reimbursing the cost of illness and reducing the economic pressure on patients. More accurate monitoring of service providers to prevent informal payments can reduce direct medical costs, especially patient payments.

## 6 CONCLUSION

Diabetes imposes a substantial economic burden on patients, health systems, and the whole economy. Besides, since the cost of the disease in patients treated with insulin and those with diabetes complications is significantly higher, the reinforcement of self‐care measures and focusing on modifying lifestyle (dietary modification and physical activity) in patients with T2DM can significantly reduce the costs of the disease.

## AUTHOR CONTRIBUTIONS

**Habib Jalilian**: conceptualization; data curation; formal analysis; methodology; project administration; software; validation; visualization; writing–original draft; writing–review & editing. **Somayeh Heydari**: writing–original draft; writing–review & editing. **Ali Imani**: supervision. **Mozhgan Salimi**: data curation. **Nazanin Mir**: data curation. **Farzad Najafipour**: project administration; supervision. All authors have read and approved the final version of the manuscript. Farzad Najafipour had full access to all of the data in this study and take complete responsibility for the integrity of the data and the accuracy of the data analysis.

## CONFLICTS OF INTEREST STATEMENT

The authors declare no conflicts of interest.

## TRANSPARENCY STATEMENT

The lead author Farzad Najafipour affirms that this manuscript is an honest, accurate, and transparent account of the study being reported; that no important aspects of the study have been omitted; and that any discrepancies from the study as planned (and, if relevant, registered) have been explained.

## Data Availability

The datasets used and/or analyzed during this study are available from the corresponding author upon reasonable request.
